# Active school commuting in adolescents from 28 countries across Africa, the Americas, and Asia: a temporal trends study

**DOI:** 10.1186/s12966-022-01404-y

**Published:** 2023-01-03

**Authors:** Mireia Felez-Nobrega, André O. Werneck, Adrian Bauman, Josep Maria Haro, Ai Koyanagi

**Affiliations:** 1 Research and Development Unit, Parc Sanitari Sant Joan de Déu, C/ Dr. Antoni Pujadas 42, 08830 Sant Boi de Llobregat, Barcelona, Spain; 2 Centre for Biomedical Research On Mental Health (CIBERSAM), ISCIII, Madrid, Spain; 3 Department of Nutrition, School of Public Health, Universidade de Sao Paulo (USP), Sao Paulo, Brazil; 4 School of Public Health, Faculty of Medicine and Health, The University of Sydney, Sydney, NSW Australia; 5 ICREA, Pg. Lluis Companys 23, Barcelona, Spain

**Keywords:** Adolescents, Longitudinal, Cohort, Active travel, Walking, Cycling

## Abstract

**Background:**

Evidence examining trends in active school commuting among adolescents are mainly single-country studies, and principally focused on high-income countries. Thus, the present study aims to examine temporal trends in adolescents’ active school commuting and to examine if there are differences in such trends by sex. We used nationally representative samples of 28 countries, which were predominantly low- and middle-income countries (LMICs), covering 5 different WHO regions.

**Methods:**

Data from the Global School-based Student Health Survey 2004–2017 were analyzed in 177,616 adolescents [mean (SD) age: 13.7 (1.0) years; 50.7% girls]. Active school commuting was self-reported (frequency of walking or riding a bike to and from school in the past 7 days). The prevalence and 95%CI of active school commuting (i.e., ≥ 3 days/week) was calculated for the overall sample and by sex for each survey. Crude linear trends in active school commuting were assessed by linear regression models. Interaction analyses were conducted to examine differing trends among boys and girls.

**Results:**

Trends in active school commuting were heterogeneous across countries, with results showing stable patterns for the majority (16/28), decreasing trends for some (7/28) and increasing trends over time for a few (5/28). The majority of countries showed no differences in active school commuting trends between girls and boys.

**Conclusions:**

The quantification of changes in adolescents’ active school commuting over time, together with a deeper understanding of local determinants for such behaviors will provide valuable evidence to inform the development of tailored and context-specific actions.

## Background

Physical activity during adolescence is a protective factor for several health outcomes, including cardiovascular risk factors, and mental disorders [1–4]. Furthermore, it is known to promote well-being, and improve quality of life and cognitive outcomes [1, 5]. However, the prevalence of physical inactivity is high worldwide, with approximately 80% of adolescents not achieving the World Health Organization (WHO) recommendations of at least an average of 60 min/day of moderate-to-vigorous physical activity [6].

Physical activity can be practiced in different domains such as leisure-time, physical education classes and transportation. In contrast to passive transport (e.g., being dropped off by a car, public transport), active commuting from/to school (ACS) (e.g., walking, biking) is an important contributor to moderate-to-vigorous physical activity [7], which represents a potential target for effective interventions aimed at increasing levels of physical activity in adolescents. Indeed, several worldwide policy actions such as the Global Action Plan on Physical Activity (GAPPA) 2018–2023 led by the World Health Organization (WHO) underline the importance of creating and supporting physical and social environments to encourage active transportation [8]. Additionally, active commuting from and to school is considered a key element of the whole-of-school approach to integrate the promotion of physical activity in schools [9].

Several complex determinants at multiple levels of influence (individual, interpersonal, organizational) are likely to be important for active commuting but compelling evidence suggest that environmental attributes (e.g., population density, urban planning, safety) are crucial for decisions on active or passive transport to school [10]. Relatedly, low- and middle-income countries (LMICs) have seen substantial changes in urban design and rapid growth in motorization, which may all affect and deprive adolescents’ opportunities for active commuting [11].

Previous multi-country and single-country studies have found that levels of active commuting to school have been stable or decreased in specific groups over the recent years [12–18]. However, most of the studies were conducted in European countries (i.e., Czech Republic, Norway, Scotland, Wales, Spain, Germany), and the few conducted in other settings are single studies in Vietnamese and Brazilian adolescents [16, 18]. Thus, comprehensive studies examining multi-country temporal trends of ACS in adolescents from other geographical areas (including LMICs) are still lacking.

A better understanding of temporal trends in ACS among adolescents is important since it can provide valuable input to help guide environmental approaches and policy decisions. These actions will ultimately have an impact on health via increases in physical activity but also by producing sustainable healthy cities (e.g., reducing the use of motorized modes of transport, contributing to reductions in the emission of greenhouse gases, leading to lower air pollution) [19]. In addition, the largest health risk of climate change is projected to occur in LMICs [20], and the creation of sustainable cities are a key contributor for combating such a planet crisis. Therefore, we aimed to analyze the time trends of ACS, and to examine if there are differences in such trends by sex. In order to provide a better understanding of global ACS trends, we used nationally representative samples of 28 under-represented countries from five-WHO defined geographical regions.

## Methods

Data from the Global School-based Student Health Survey (GSHS) were analyzed (survey details and publicly available data can be found at: https://www.cdc.gov/gshs/index.htm). In brief, the GSHS was developed by the World Health Organization, the US Centers for Disease Control and Prevention (CDC), and other UN allies. The survey is based on the CDC Youth Risk Behavior Survey (YRBS) for which test–retest reliability has been established [21]. The survey used a standardized two-stage probability sampling design for the selection process within each participating country. First, schools were selected with probability proportional to size sampling. The second stage involved the random selection of classrooms which included students aged 13–15 years within each selected school. Irrespective of age, all students in the selected classrooms were eligible to participate in the survey and data collection was conducted during one regular class period. The questionnaire was translated into the local language in each country and students recorded their response on computer scannable sheets. A national government administration (most often the Ministry of Health or Education) and an institutional review board/ethics committee approved GSHS surveys in each country. The participation was anonymous and voluntary, and informed consent was obtained as appropriate from the students, parents and/or school officials. Data were weighted for non-response and probability selection.

From all publicly available data, we selected all nationally representative datasets that included the variables used in the current analysis, and countries for which data on at least two waves were available. Thus, a total of 28 countries were included in the current study. The characteristics of each country or survey are provided in Table 1. For the included countries, the survey was conducted between 2004 and 2017.

**Table 1:** Survey characteristics

Region	Country	Country income	Year	Response rate (%)	N^a^	Females (%)
AFR	Benin	L	2009	90	1,170	33.9
		L	2016	78	717	34.4
	Mauritius	UM	2007	88	1,961	53.2
		UM	2011	82	2,074	50.8
		UM	2017	84	1,955	54.2
	Namibia	LM	2004	82	4,529	56.9
		UM	2013	89	1,936	57.1
	Seychelles	UM	2007	82	1,154	50.1
		H	2015	82	2,061	50.5
AMR	Argentina	UM	2007	77	1,537	54.3
		UM	2012	71	21,528	52.3
	Guatemala	LM	2009	81	4,495	47.8
		LM	2015	82	3,611	49.1
	Guyana	LM	2004	80	1,070	52.9
		LM	2010	76	1,973	51.4
	Suriname	UM	2009	89	1,046	54.6
		UM	2016	83	1,453	53.9
	Trinidad & Tobago	H	2007	78	2,450	50.9
		H	2011	90	2,363	50.5
		H	2017	89	2,763	51.7
	Uruguay	UM	2006	71	2,882	54.9
		H	2012	77	2,869	53.7
EMR	Egypt	LM	2006	87	4,981	48.2
		LM	2011	85	2,364	50.8
	Jordan	LM	2004	95	1,848	51.6
		LM	2007	99.8	1,648	52.7
	Kuwait	H	2011	85	2,298	48.7
		H	2015	78	2,034	50.6
	Lebanon	UM	2011	87	1,982	53.4
		UM	2017	82	3,347	52.6
	Morocco	LM	2006	84	1,986	47.2
		LM	2010	92	2,405	47.1
		LM	2016	91	3,975	49.1
	Oman	UM	2005	97	2,426	48.5
		H	2010	89	1,000	52.0
		H	2015	92	1,669	52.9
	UAE	H	2005	89	12,819	51.6
		H	2010	91	2,302	60.1
		H	2016	80	3,471	51.9
	Yemen	L	2008	82	905	34.4
		LM	2014	75	1,553	43.7
SEAR	Indonesia	LM	2007	93	3,022	50.7
		LM	2015	94	8,806	50.8
	Myanmar	L	2007	95	2,227	50.5
		LM	2016	86	2,237	53.7
	Sri Lanka	LM	2008	89	2,504	50.2
		LM	2016	89	2,254	50.7
	Thailand	LM	2008	93	2,675	51.6
		UM	2015	89	4,132	50.4
WPR	Cook Islands	-	2011	84	849	47.4
		-	2015	65	366	51.3
	Fiji	LM	2010	90	1,495	51.4
		UM	2016	79	1,537	51.0
	Philippines	LM	2007	81	3,484	55.4
		LM	2011	82	3,845	51.5
		LM	2015	79	6,162	51.9
	Samoa	LM	2011	79	2,200	52.6
		LM	2017	59	1,058	53.6
	Tonga	LM	2010	80	1,946	49.7
		UM	2017	90	2,067	48.6
	Vanuatu	LM	2011	72	852	50.5
		LM	2016	57	1,288	52.2

*Abbreviation*: *AFR* African Region, *AMR* Region of the Americas, *EMR* Eastern Mediterranean Region, *SEAR* South-East Asia Region, *WPR* Western Pacific Region, *UAE* United Arab Emirates, *H* high income, *L* low income, *LM* lower middle-income, *UM* upper middle-income. No data on country income level is available for Cook Islands

^a^Based on sample aged 12–15 years

### Sex and active commuting from/to school

Sex (male or female) was self-reported. ACS was assessed with one item: ‘During the past 7 days, on how many days did you walk or ride a bicycle to and from school?’ The response options were 0–7 days. Similar to previous research, we classified students as ‘active school commuters’ if they rode a bicycle or walked to and from school on ≥ 3 days during the previous 7 days [22–24].

### Statistical analysis

Statistical analyses were performed with Stata 14.1 (Stata Corp LP, College station, Texas). The analysis was restricted to those aged 12–15 years as most students were within this age group, while information on the exact age outside of this age range was not available. The prevalence and 95%CI of ACS (i.e., ≥ 3 days/week) was calculated for the overall sample and by sex for each survey. Crude linear trends in ACS were assessed by linear regression models across surveys within the same country to estimate regression coefficients (beta) and 95%CI for every one-year change. P for trends were estimated using the survey year as a continuous variable. The beta can be interpreted as the average percentage point change in prevalence per year. We also conducted interaction analysis to assess whether there are differing trends among boys and girls by including an interaction term (survey year X sex) in the model. Sampling weights and the clustered sampling design of the surveys were taken into account in all analyses.

## Results

**Fig. 1:**
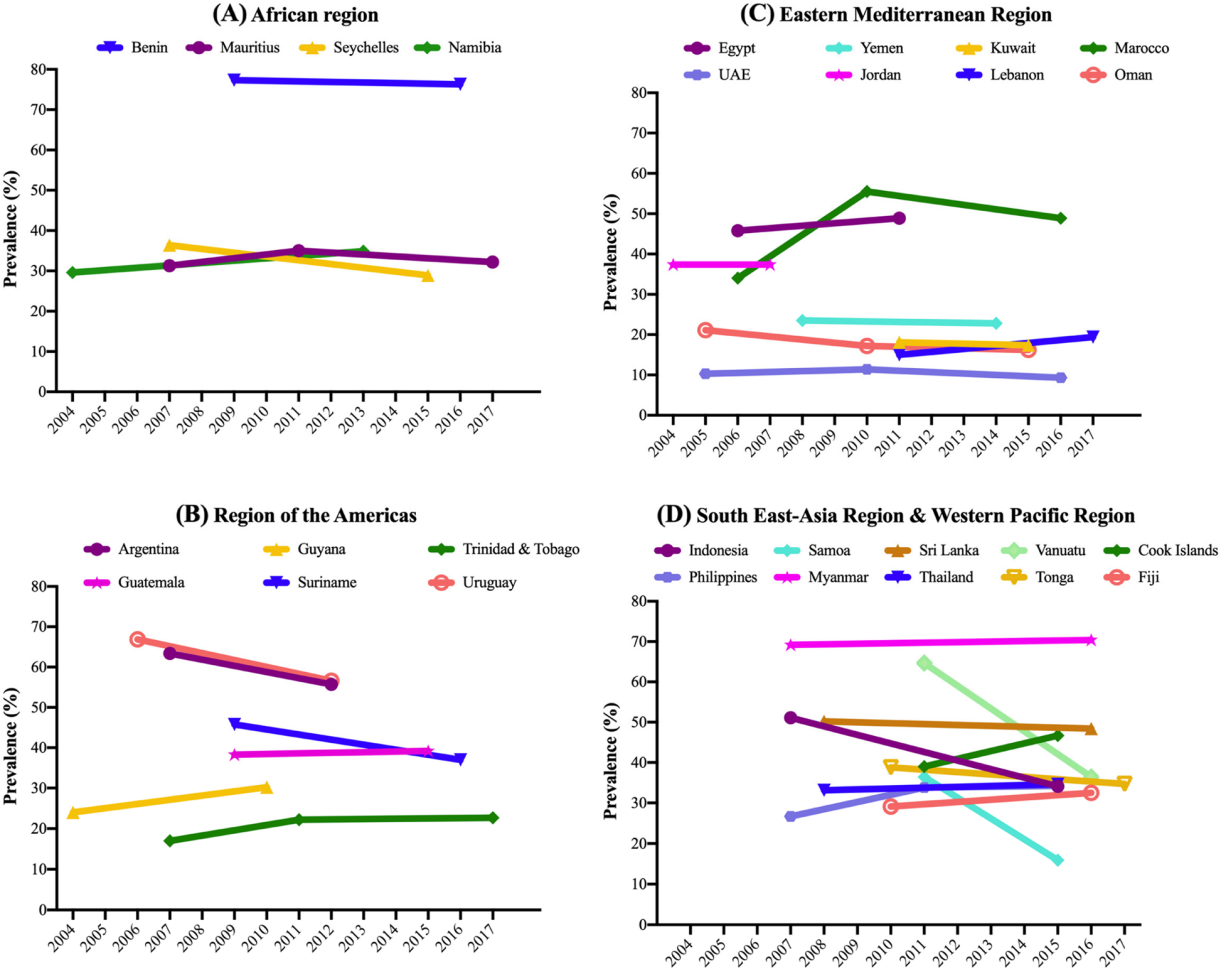
Trends in prevalence (%) of ACS (≥ 3 days/week) by country and region (overall)

**Fig. 2:**
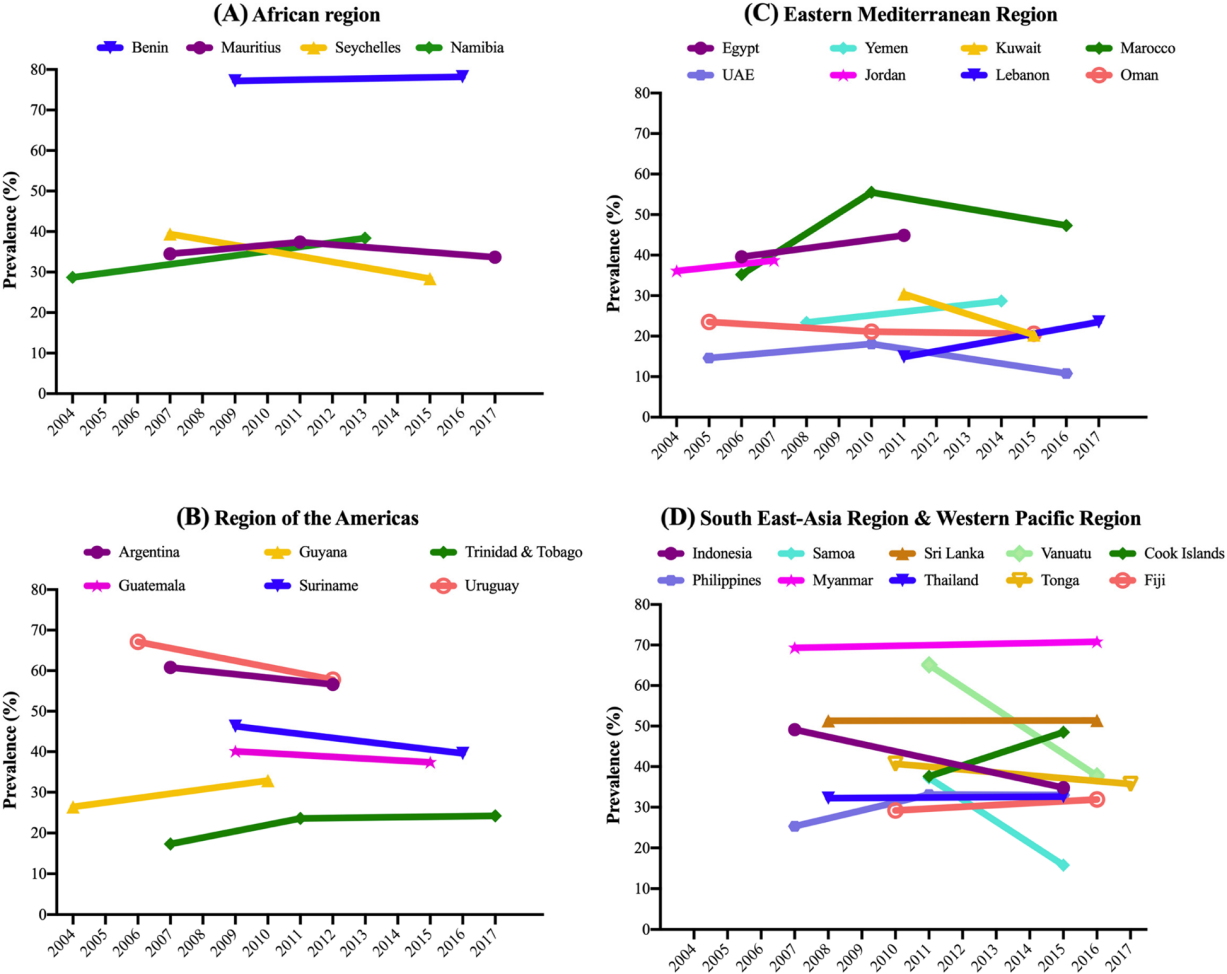
Trends in prevalence (%) of ACS (≥ 3 days/week) by country and region (boys)

**Fig. 3:**
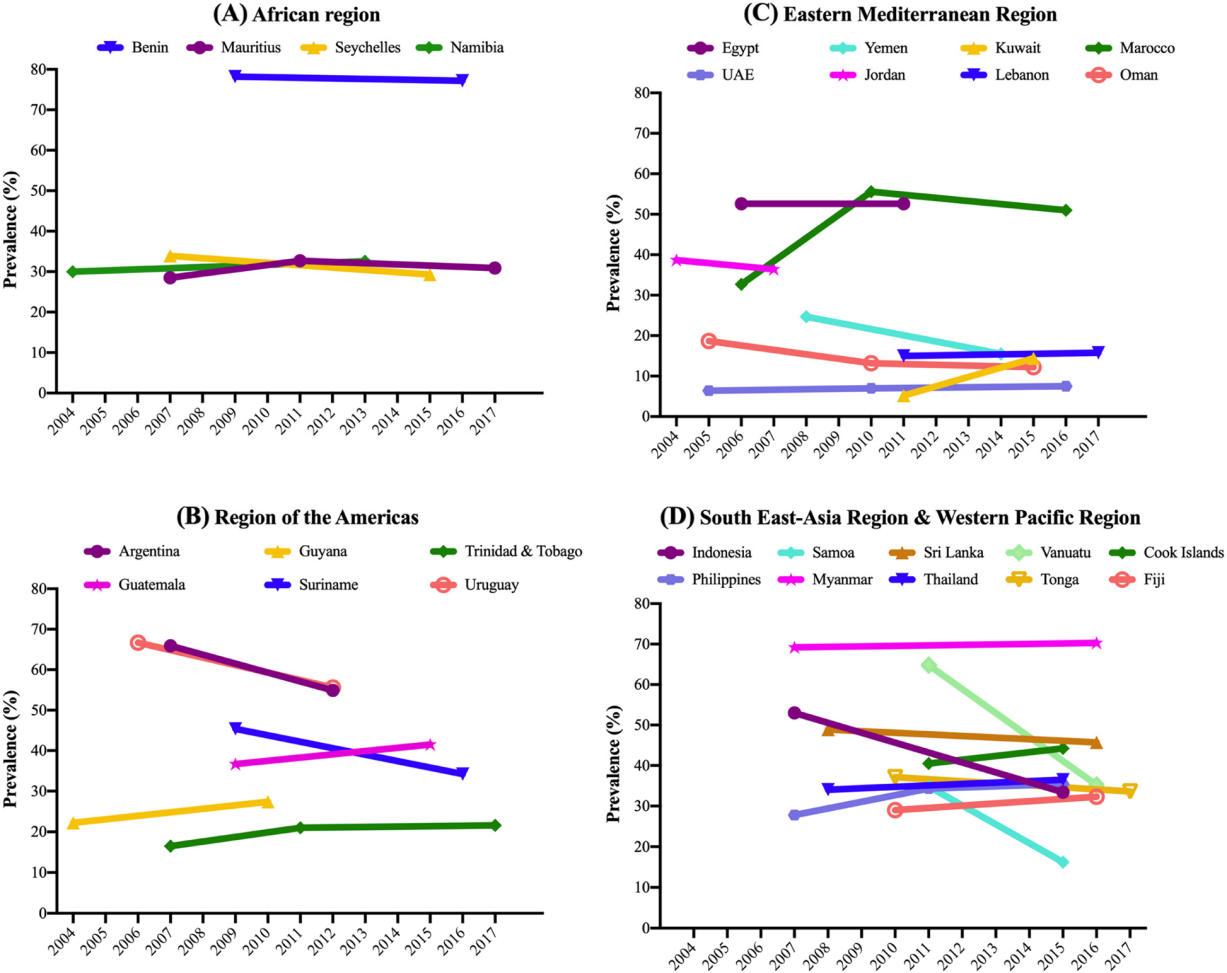
Trends in prevalence (%) of ACS (≥ 3 days/week) by country and region (girls)

Data on 177,616 students aged 12–15 years [mean (SD) age 13.7 (1.0) years; 50.7% girls] were used for the current analysis. The trends in ACS are shown in Table 2, Fig. 1 (overall), Fig. 2 (boys), and Fig. 3 (girls). For the overall sample including both boys and girls, the prevalence of ACS (i.e., ≥ 3 days/week) was 40.3% (41.0% for girls; and 39.6% for boys). The prevalence ranged from 9.3% in United Arab Emirates (2016) to 77.3% in Benin (2009).

**Table 2:** Trends in prevalence (%) of high levels of active school commuting (≥ 3 days/week) in 28 countries (overall and by sex)

		Overall			Boys			Girls		
Country	Year	% [95%CI]	beta^a^ [95%CI]	p for trend^a^	% [95%CI]	beta^a^ [95%CI]	p for trend^a^	% [95%CI]	beta^a^ [95%CI]	p for trend^a^
**AFR**										
Benin	2009	77.3 [73.7,80.6]	-0.15 [-1.14,0.84]	0.758	77.2 [74.6,79.7]	-0.21 [-1.10,0.69]	0.641	78.2 [70.3,84.5]	-0.15 [-1.67,1.36]	0.839
	2016	76.3 [70.0,81.5]			75.8 [69.9,80.8]			77.2 [68.9,83.7]		
Mauritius	2007	31.3 [26.8,36.2]	0.03 [-0.56,0.61]	0.923	34.5 [31.2,37.9]	-0.13 [-0.75,0.48]	0.662	28.5 [21.7,36.5]	0.18 [-0.63,0.99]	0.660
	2011	35.0 [30.5,39.8]			37.4 [32.1,43.1]			32.7 [26.6,39.4]		
	2017	32.2 [28.9,35.8]			33.7 [29.0,38.8]			30.9 [27.2,34.9]		
Namibia	2004	29.6 [26.7,32.6]	0.60 [0.04,1.15]	0.036	28.7 [25.7,31.8]	1.08 [0.47,1.69]	0.001	30.0 [26.2,34.2]	0.28 [-0.40,0.97]	0.414
	2013	34.9 [31.1,39.0]			38.4 [34.0,42.9]			32.6 [28.2,37.3]		
Seychelles	2007	36.4 [35.2,37.6]	-0.93 [-1.28,-0.58]	< 0.001	39.4 [37.3,41.5]	-1.37 [-1.86,-0.88]	< 0.001	33.9 [33.0,34.8]	-0.57 [-1.00,-0.13]	0.011
	2015	28.9 [26.5,31.5]			28.4 [25.3,31.9]			29.3 [26.1,32.8]		
**AMR**										
Argentina	2007	63.4 [55.4,70.7]	-1.55 [-3.20,0.10]	0.066	60.8 [52.6,68.4]	-0.84 [-2.58,0.91]	0.345	65.9 [57.4,73.5]	-2.20 [-3.95,-0.45]	0.014
	2012	55.7 [52.8,58.5]			56.6 [53.1,60.0]			54.9 [51.7,58.1]		
Guatemala	2009	38.3 [34.9,41.8]	0.15 [-0.76,1.06]	0.745	40.1 [34.8,45.5]	-0.45 [-1.71,0.81]	0.482	36.7 [32.6,41.0]	0.79 [-0.35,1.93]	0.171
	2015	39.2 [35.1,43.4]			37.4 [32.4,42.7]			41.5 [36.3,46.8]		
Guyana	2004	24.0 [20.3,28.3]	1.02 [-0.10,2.15]	0.074	26.4 [21.5,32.0]	1.08 [-0.34,2.51]	0.130	22.2 [17.7,27.6]	0.86 [-0.40,2.12]	0.175
	2010	30.2 [25.3,35.5]			32.9 [27.0,39.5]			27.4 [22.4,33.0]		
Suriname	2009	45.8 [39.0,52.7]	-1.26 [-2.64,0.12]	0.071	46.3 [39.7,53.0]	-0.95 [-2.42,0.52]	0.196	45.4 [37.4,53.8]	-1.61 [-3.19,-0.03]	0.047
	2016	37.0 [31.1,43.3]			39.6 [32.7,47.0]			34.2 [28.0,41.0]		
Trinidad & Tobago	2007	17.0 [14.7,19.7]	0.55 [0.16,0.93]	0.006	17.3 [13.2,22.4]	0.66 [0.05,1.26]	0.033	16.5 [13.1,20.5]	0.49 [-0.06,1.04]	0.080
	2011	22.2 [19.1,25.8]			23.6 [20.0,27.7]			21.0 [16.5,26.3]		
	2017	22.7 [20.0,25.7]			24.2 [20.7,28.0]			21.6 [17.9,25.9]		
Uruguay	2006	66.9 [63.7,69.9]	-1.71 [-2.72,-0.69]	0.001	67.1 [63.5,70.4]	-1.55 [-2.65,-0.44]	0.007	66.7 [63.0,70.2]	-1.85 [-3.00,-0.70]	0.002
	2012	56.6 [51.4,61.7]			57.8 [52.2,63.2]			55.6 [49.8,61.3]		
**EMR**										
Egypt	2006	45.8 [36.9,55.0]	0.63 [-1.71,2.96]	0.593	39.6 [31.5,48.3]	1.06 [-1.47,3.58]	0.404	52.6 [40.9,64.0]	0.01 [-3.06,3.08]	0.996
	2011	48.9 [42.1,55.8]			44.9 [36.2,53.9]			52.6 [43.4,61.7]		
Jordan	2004	37.4 [34.1,40.9]	-0.00 [-1.88,1.88]	1.000	36.1 [31.1,41.3]	0.87 [-1.14,2.87]	0.379	38.7 [34.9,42.6]	-0.78 [-3.75,2.18]	0.593
	2007	37.4 [33.4,41.7]			38.7 [36.2,41.3]			36.4 [29.2,44.2]		
Kuwait	2011	18.1 [15.5,21.1]	-0.17 [-1.82,1.47]	0.830	30.4 [27.0,34.0]	-2.52 [-4.24,-0.81]	0.006	5.2 [3.8,7.0]	2.31 [0.69,3.94]	0.007
	2015	17.4 [12.5,23.8]			20.3 [15.3,26.4]			14.4 [9.4,21.5]		
Lebanon	2011	15.0 [9.9,22.0]	0.73 [-0.35,1.81]	0.177	14.9 [9.7,22.2]	1.44 [0.28,2.59]	0.016	15.0 [9.6,22.8]	0.13 [-1.07,1.33]	0.831
	2017	19.4 [17.5,21.3]			23.5 [21.0,26.1]			15.8 [13.5,18.4]		
Morocco	2006	34.0 [29.9,38.3]	1.15 [0.53,1.78]	0.001	35.2 [30.6,40.0]	0.88 [0.29,1.47]	0.004	32.7 [28.3,37.5]	1.47 [0.64,2.29]	0.001
	2010	55.5 [52.1,58.8]			55.5 [52.5,58.5]			55.6 [49.8,61.2]		
	2016	48.9 [44.6,53.4]			47.3 [43.8,50.9]			51.0 [44.7,57.3]		
Oman	2005	21.1 [19.2,23.2]	-0.51 [-0.86,-0.16]	0.004	23.5 [20.8,26.4]	-0.30 [-0.80,0.19]	0.225	18.7 [15.3,22.7]	-0.67 [-1.14,-0.21]	0.005
	2010	17.2 [14.0,20.9]			21.1 [17.8,24.8]			13.2 [9.6,18.0]		
	2015	16.2 [13.6,19.2]			20.6 [16.8,25.0]			12.2 [9.9,15.0]		
UAE	2005	10.3 [8.7, 12.2]	-0.11 [-0.36,0.15]	0.407	14.6 [12.6,16.8]	-0.38 [-0.65,-0.11]	0.007	6.4 [4.6,8.8]	0.10 [-0.21,0.41]	0.512
	2010	11.4 [8.7,14.7]			18.1 [13.3,24.2]			7.0 [5.5,8.9]		
	2016	9.3 [7.4,11.5]			10.8 [9.0,13.0]			7.5 [5.3,10.6]		
Yemen	2008	23.5 [17.5,30.7]	-0.11 [-1.55,1.34]	0.882	23.4 [17.7,30.3]	0.88 [-0.76,2.52]	0.281	24.7 [12.7,42.6]	-1.54 [-4.26,1.18]	0.257
	2014	22.8 [18.2,28.2]			28.7 [22.2,36.1]			15.5 [12.2,19.5]		
**SEAR**										
Indonesia	2007	51.1 [44.2,57.9]	-2.13 [-3.29,-0.96]	0.001	49.1[41.6,56.6]	-1.79 [-3.02,-0.56]	0.005	53.0 [45.9,60.0]	-2.45 [-3.66,-1.25]	< 0.001
	2015	34.1 [28.3,40.4]			34.8 [29.1,41.0]			33.4 [27.4,40.0]		
Myanmar	2007	69.2 [63.8,74.2]	0.13 [-0.62,0.88]	0.730	69.3 [63.7,74.3]	0.17 [-0.57,0.91]	0.645	69.2 [62.8,75.0]	0.12 [-0.82,1.06]	0.798
	2016	70.4 [66.2,74.3]			70.8 [66.8,74.5]			70.3 [64.5,75.5]		
Sri Lanka	2008	50.2 [46.5,53.8]	-0.22 [-0.97,0.52]	0.551	51.3 [46.0,56.5]	0.01 [-0.93,0.95]	0.984	48.9 [44.3,53.5]	-0.41 [-1.55,0.73]	0.472
	2016	48.4 [43.9,52.9]			51.4 [46.4,56.3]			45.7 [38.3,53.2]		
Thailand	2008	33.1 [29.0,37.5]	0.21 [-0.52,0.95]	0.562	32.2 [26.9,37.8]	0.07 [-0.87,1.00]	0.887	34.0 [29.6,38.7]	0.36 [-0.53,1.25]	0.420
	2015	34.6 [32.0,37.3]			32.6 [29.4,36.0]			36.5 [32.6,40.5]		
**WPR**										
Cook Islands	2011	39.0 [39.0,39.0]	1.94 [0.31,3.56]	0.020	37.6 [37.6,37.6]	2.73 [0.38,5.08]	0.024	40.5 [40.5,40.5]	0.94 [-0.83,2.71]	0.291
	2015	46.7 [40.5,53.1]			48.5 [39.6,57.6]			44.2 [37.5,51.2]		
Fiji	2010	29.1 [25.1,33.4]	0.57 [-0.42,1.56]	0.245	29.2 [25.2,33.6]	0.44 [-0.68,1.56]	0.427	29.0 [24.3,34.2]	0.54 [-0.52,1.61]	0.305
	2016	32.5 [28.8,36.5]			31.9 [27.2,36.9]			32.3 [28.7,36.0]		
Philippines	2007	26.7 [22.5,31.3]	0.82 [0.03,1.61	0.043	25.3 [20.5,30.8]	0.80 [-0.080,1.67]	0.075	27.8 [23.4,32.7]	0.86 [0.01, 1.70]	0.047
	2011	33.8 [28.4,39.7]			33.1 [26.1,41.1]			34.3 [29.9,38.9]		
	2015	34.2 [30.2,38.4]			33.0 [29.0,37.2]			35.4 [30.9,40.1]		
Samoa	2011	36.4 [34.3,38.5]	-3.41 [-4.11,-2.72]	< 0.001	37.2 [33.6,40.9]	-3.56 [-4.68,-2.45]	< 0.001	34.8 [32.8,36.7]	-3.09 [-3.69,-2.50]	< 0.001
	2017	15.9 [12.7,19.7]			15.8 [11.1,22.0]			16.2 [13.5,19.3]		
Tonga	2010	38.8 [35.7,41.9]	-0.58 [-1.14,-0.01]	0.045	40.7 [36.0,45.7]	-0.72 [-1.56,0.12]	0.091	37.1 [33.6,40.8]	-0.50 [-1.19,0.19]	0.155
	2017	34.7 [32.4,37.2]			35.7 [32.5,39.0]			33.6 [30.5,36.8]		
Vanuatu	2011	64.7 [50.4,76.8]	-5.70 [-6.54,-4.87]	< 0.001	65.1 [51.5,76.5]	-5.82 [-7.13, -4.51]	< 0.001	64.8 [48.1,78.4]	-5.67 [-6.77, -4.58]	< 0.001
	2016	36.4 [32.4,40.5]			37.7 [31.7,44.1]			35.2 [29.9,40.9]		

*Abbreviation*: *CI* Confidence interval, *AFR* African Region, *AMR* Region of the Americas, *EMR* Eastern Mediterranean Region, *SEAR* South-East Asia Region, *WPR* Western Pacific Region, *UAE* United Arab Emirates

^a^The beta and P for trend are based on linear regression including survey year as a continuous variable. The beta can be interpreted as the average percentage point change in prevalence per year

Overall, significant increasing trends for ACS were observed in Namibia between 2004 (29.6%) and 2013 (34.9%) (beta = 0.60; 95%CI = 0.04,1.15), Trinidad & Tobago between 2007 (17.0%) and 2017 (22.7%) (beta = 0.55; 95%CI = 0.16,0.93), Morocco between 2006 (34%) and 2016 (48.9%) (beta = 1.15; 95%CI = 0.53,1.78), Cook Islands between 2011 (39.0%) and 2015 (46.7%) (beta = 1.94; 95%CI = 0.31,3.56), and Philippines between 2007 (26.7%) and 2015 (34.2%) (beta = 0.82; 95%CI = 0.03,1.61). Conversely, significant decreasing trends in ACS were found in Seychelles between 2007 (36.4%) and 2015 (28.9%) (beta = -0.93; 95%CI = -1.28,-0.58), Uruguay between 2006 (66.9%) and 2012 (56.6%) (beta = -1.71; 95%CI = -2.72,-0.69), Oman between 2005 (21.1%) and 2015 (16.2%) (beta = -0.51; 95%CI = -0.86,-0.16), Indonesia between 2007 (51.1%) and 2015 (34.1%) (beta = -2.13; 95%CI = -3.29,-0.96), Samoa between 2011 (36.4%) and 2017 (15.9%) (beta = -3.41; 95%CI = -4.11,-2.72), Tonga between 2010 (38.8%) and 2017 (34.7%) (beta = -0.58; 95%CI = -1.14,-0.01), and Vanuatu between 2011 (64.7%) and 2016 (36.4%) (beta = -5.70; 95%CI = -6.54,-4.87). For the rest of countries, the overall prevalence of ACS remained stable over time.

While the majority of the countries showed similar trends between girls and boys, significant sex-differences were found in the interaction analysis for six countries (Namibia, Seychelles, Argentina, Kuwait, Lebanon, and United Arab Emirates). In Namibia and Lebanon, a significant increase in ACS was only observed among boys (Namibia, 2004–2013; beta = 1.08; 95%CI = 0.47,1.69; Lebanon, 2011–2017; beta = 1.44; 95%CI = 0.28,2.59). In Argentina, the decrease was only significant in girls (2007–2012; beta = -2.20; 95%CI = -3.95,-0.45), while in United Arab Emirates, it was significant only in boys (2005–2016; beta = -0.38, 95%CI = -0.65,-0.11). In Kuwait, complete opposite trends between sexes were found, with boys showing significant decreasing trends (2011–2015; beta = -2.52; 95%CI = -4.24,-0.81) and girls significant increases (beta = 2.31; 95%CI = 0.69,3.94). In Seychelles, the significant interaction was driven by more pronounced decreases in ACS between 2007 and 2015 among boys (beta = -1.37; 95%CI = -1.86,-0.88).

## Discussion

This study aimed to examine trends in adolescents’ ACS over time in 28 countries from 5 WHO regions in Africa, the Americas, and Asia. Included countries were predominantly LMICs, and the need to increase physical activity research in LMICs is an established research gap in the existing literature [5, 25].

Our study results showed that ACS is not a homogeneous phenomenon across countries. Indeed, we found stable patterns over time for the majority of countries (16 out of 28), decreasing trends for some countries (7/28), and increasing trends for a few (5/28). Time trend studies are scarce in LMICs, but our results are not completely in line with the few that are available since they consistently show decreasing trends in ACS over time (Brazil, between 2005 to 2012 [16]; Vietnam, 2004–2017 [18]; China, 1997–2006 [26]. Notwithstanding, caution is urged when interpreting such comparisons since distinct operationalization of ACS variables and research methodologies are present (e.g., the number of time points available in the previous studies ranged from 2 to 5).

Several factors may potentially explain the decreasing trends in ACS found for some countries. First, it is well documented that LMICs are experiencing rapid socioeconomic development and growth in motorized vehicles [27]. In addition, demographic transitions are occurring rapidly in LMICs with people moving from rural to urban areas [28], and it has been suggested that people living in urban areas are more likely to use passive transportation [16, 18, 26, 29]. In addition, it may be hypothesized that the rapid urban sprawl, and sub-urbanization may have increased distance to school in some settings, or specialized or preferred schools are at increasing distance from where people live. Research conducted in high-income countries identified distance to school as a main barrier to ACS [11], and showed that living in sub-urban areas has limited people’s ability to actively commute and reinforced car-dependency [30, 31].

The present study also found stable patterns in ACS for most of the included countries across different regions. It is possible that the aforementioned social and economic megatrends that affect society have occurred at a much more rapid speed in the past but have been relatively stable over the past fifteen years, which may partially explain such steady prevalence in ACS over time observed in the majority of the countries in our study. For instance, previous studies based on industrialized countries (Canada, Australia, US, Switzerland) showed that declines in ACS occurred over more than three decades ago (1986–2006) [32–36], but more recent studies (2006–2018) based in Europe suggest that these patterns remained stable [12, 13], (with the exception of Germany and Czech Republic that reported decreasing trends from 2003 to 2017, and between 2006 and 2010, respectively) [12, 17]. Alternatively, it is possible that the societal megatrends do not directly shape ACS over time, but that this behavior might be triggered automatically and strongly underpinned by habits (behaviors repeated regularly with little or no conscious thought) that are difficult to modify [37]. This, together with the fact that the promotion of active transportation is usually neglected from a public health perspective and receives scarce attention from local governments and authorities may contribute to the stable patterns found in the majority of the countries.

Importantly, several countries reported very low levels of ACS with less than a quarter of their adolescents engaging in ACS on at least 3 days/week. In addition, it should be highlighted that the low ACS levels remained stable (United Arab Emirates, Kuwait, Lebanon) or even worsened over time (Oman). Weather may be an important factor that influences travel behaviors, since such countries were all located in the Eastern Mediterranean Region. This region is characterized by persistent hot and dry weather conditions (especially during summer), and the aforementioned countries also have high levels of vehicle ownership [38], which may explain in part the low levels of ACS found in the present study. Efforts towards designing and testing tailored and contextually adapted interventions to increase ACS in such contexts is needed. Unfortunately, little is known about moderators and mediators that influence travel behavior change, in this population. Relatedly, research in this area is in its infancy and previous intervention studies to increase ACS from high-income settings (US, Europe, Australia) reported small effect sizes and the quality of evidence was weak [39, 40].

Our study also revealed increasing trends in ACS for a few countries (i.e., Namibia, Trinidad & Tobago, Morocco, Cook Islands, and Philippines). While the reason for this trend is only speculative, it is possible that with rapid urbanization occurring in some settings, particularly LMICs, vulnerable populations (i.e., socioeconomically disadvantaged) have settled in the periphery of large cities, with perhaps no schools in the local community, which force adolescents to actively commute to/from school. Alternatively, economic development may increase the number of schools for some countries or urban contexts, and the higher availability of schools located nearby may have shortened distance to schools and fostered active commuting choices. In addition, low affordability of public transportation and potential increases in fares may be obstacles for low-income urban residents to use this mode of transportation, and they may have opted for alternative and cheaper modes to travel to school such as active commuting. It is also possible that there has been a more widespread access to education for rural communities in LMICs, which could also have contributed to increases in ACS over time.

Finally, we found that most countries showed no differences in ACS trends between boys and girls, which is in line with previous findings in Brazilian and German adolescents [16, 17]. However, in the current study, differences in ACS over time between boys and girls were apparent in some countries. Specifically, girls in Argentina showed decreases in ACS while the trend for boys remained stable. In Namibia and Lebanon, trends did not change in girls and only significant increases were observed for boys. Several reported barriers by parents may possibly explain in part such decreasing or stable patterns for girls, including concerns related to perceived traffic, personal safety concerns (e.g., violence, rape, harassment, or crime safety) [41–43].

In United Arab Emirates, Seychelles and Kuwait, abrupt decreases occurred in boys, while significant increases in ACS were found for girls in Kuwait (in United Arab Emirates and Seychelles, this remained stable). Cultural/religious barriers to overall physical activity have been previously documented in females from Arab countries (e.g., lack of encouragement, conservative clothing not suitable for physical activity, time constraints from academic/family responsibilities, being in public spaces accompanied, paucity of gender segregated facilities, etc..) [44, 45]. Indeed, in our study, we observed notable differences in the prevalence of ACS among girls and boys in some countries (e.g., Kuwait in 2011, Oman in 2015, United Arab Emirates in 2010), where girls were much less likely to engage in ACS. Given that these were all Arab or Mainly Muslim countries, it is possible that there are religious and cultural barriers for girls to engage in ACS. However, it seems that these factors are unlikely to explain the observed trends, since we found significant increasing or stable trends for girls in United Arab Emirates, Seychelles and Kuwait. Other factors such as the political stand, changes in societal/gender norms and potential changes in women’s rights have contributed to increasing or stable ACS for girls [46]. Importantly, given the limited research on this topic, the aforementioned hypotheses are speculative in nature, and clearly, more research is warranted on the correlates and determinants of travel behaviors according to sex/gender in different settings.

The promotion of physical activity was previously based on theoretical approaches that primarily target personal level factors. However, psychosocial and environmental variables best explain physical activity behavior [47]. Indeed, it is well known that physical activity − and ACS in particular − , are behaviors that are influenced by a complex interplay of personal, behavioral, social/psychological and environmental factors [47]. Thus, the prioritization of environmental rather than individual approaches for physical activity promotion has already been advocated [48]. Recently, the Lancet has published the second Series on urban design, transport, and health which is pioneer work on how to facilitate the creation of worldwide sustainable cities that encourage urban and population health [49]. Importantly, research on city planning and capacity building in LMICs is a current research gap that should guide future research endeavors [49].

Importantly, ACS is considered a sustainable behavior that has positive consequences for the health of individuals and societies, but it may also entail important safety risks. LMICs experience 90% of the worlds’ traffic fatalities and injuries [50], and pedestrians and cyclists are the group that is most vulnerable to such events [51]. In addition, several studies conducted in developing countries suggest that those with lower socioeconomic backgrounds (e.g., living in rural or suburban areas, least wealthy families, lower socio-economic schools) are more prone to use active transportation [16, 18, 26, 29]. The overall lack of public transportation infrastructure in rural areas, schools located further, and the low car ownership rates for the poorest segments of the population may force the adolescents to actively commute to/from school. Indeed, socioeconomic determinants not only play an important role and directly influence overall travel behaviors, but they foster increased vulnerability to road traffic injuries. Additionally, the efforts to improve ACS in adolescence should also take into consideration safe environments, especially among girls, in which they need to feel secure. Altogether, this highlights the need for protecting such users by providing safe and high-quality infrastructure to promote ACS and overall active commuting behaviors.

This study contributed to building a stronger evidence base and to expand physical activity-related surveillance in under-represented countries. The large sample size and the use of standardized comparable measures of ACS allowed for direct comparisons across countries. Nonetheless, the present findings should be interpreted in light of several limitations. First, there is no gold standard or established consensus related to ACS measurement. In addition, the present study is based on active commuting to school, but active commuting purposes for other day-to-day activities may potentially be important. Second, similar to previous studies conducted on the topic, the climate linked to the timing of data collection may have influenced the ACS estimates since weather conditions may affect transportation choices (e.g., rainy season). Finally, not all surveys were conducted in the same years for all the countries which makes the estimates not entirely comparable across countries. Relatedly, there were some cases where the time frames do not overlap. For example, the timeframe of Jordan was between 2004 and 2007, while in Kuwait, Lebanon, Cook Islands, Samoa, and Vanuatu, the timeframe was from 2011. Thus, data should always be interpreted in conjunction with the year in which the surveys were conducted. Further, temporal trends are more accurate in the countries that provided more than two datapoints.

## Conclusion

In conclusion, we found great regional variation in temporal trends of ACS, which highlights the importance of providing local evidence for guiding and planning policy decisions. The quantification of changes in adolescents’ ACS over time, together with a deeper understanding of local determinants for such behaviors will provide valuable evidence to inform the development of tailored and context-specific actions.

## Data Availability

The dataset supporting the conclusions of this article is available in: https://www.cdc.gov/gshs/index.htm

## Abbreviations

ACS: Active commuting from/to school
LMICs: Low- and middle-income countries
GSHS: Global School-based Student Health Survey
CDC: Centers for Disease Control and Prevention
YRBS: Youth Risk Behavior Survey
